# Differences in phenological term changes in field crops and wild plants – do they have the same response to climate change in Central Europe?

**DOI:** 10.1007/s00484-024-02846-8

**Published:** 2025-01-07

**Authors:** L Bartošová, L Hájková, E Pohanková, M Možný, J Balek, P Zahradníček, P Štěpánek, P Dížková, M Trnka, Z Žalud

**Affiliations:** 1 Department of Climate Change Impacts on Agroecosystems, Institute of Global Change Research of the Academy of Sciences of the Czech Republic, Bělidla 986/4b, Brno, 60300 Czech Republic; 2 Department of Agrosystems and Bioclimatology, Faculty of AgriSciences, Mendel University in Brno, Zemědělská 1, Brno, Czech Republic; 3 Department of Biometeorological Applications, Czech Hydrometeorological Institute, Na Šabatce 17, Prague, Czech Republic

**Keywords:** phenology, trends, asynchrony, crop plants, wild plants

## Abstract

Phenological shifts in wild-growing plants and wild animal phenophases are well documented at many European sites. Less is known about phenological shifts in agricultural plants and how wild ecosystem phenology interacts with crop phenology. Here, we present long-term phenological observations (1961–2021) from the Czech Republic for wild plants and agricultural crops and how the timing of phenophases differs from each other. The phenology of wild-growing plants was observed at various experimental sites with no agriculture or forestry management within the Czech Hydrometeorological Institute observations. The phenological data of the crops were collected from small experimental plots at the Central Institute for Supervising and Testing in Agriculture. The data clearly show a tendency to shift to earlier times during the observation period. The data also show some asynchrony in phenological shifts. Compared with wild plants, agricultural crops showed more expressive shifts to the start of the season. Phenological trends for crop plants (*Triticum aestivum*) showed accelerated shifts of 4.1 and 5.1 days per decade at low and middle altitudes, respectively; on the other hand, the average phenological shift for wild plants showed smaller shifts of 2.7 and 2.9 days per decade at low and middle altitudes, respectively. The phenophase ´heading´ of *T. aestivum* showed the highest correlation with maximum temperatures (*r* = 0.9), followed by wild species (with *r* = 0.7–0.8) and two remaining phenophases of *T. aestivum* jointing and ripening (with *r* = 0.7 and 0.6). To better understand the impacts of climate on phenological changes, it is optimal to evaluate natural and unaffected plant responses in wild species since the phenology of field crops is most probably influenced not only by climate but also by agricultural management.

**Supplementary information:**

The online version contains supplementary material available at 10.1007/s00484-024-02846-8.

## Introduction

Phenology—the timing of the seasonal activities of animals and plants—is defined as perhaps the simplest process by which to track changes in the ecology of species in response to climate change by the Intergovernmental Panel on Climate Change (IPCC) in 2007 (Rosenzweig et al. 2007). This description of phenology follows a high number of papers (published in the 1990s and at the turn of the millennium) and their conclusions about the shifting of phenophases to earlier times and the connection between the timing of phenophases and climate, mainly temperature for plants (e.g., Menzel 2000; Menzel and Estrella 2001) and for animals (e.g., Beebee 1995; Crick et al. 1997; Roy and Sparks 2000). During the first decade of the 21st century, several papers were published, mainly about the phenological differences and similarities among communities (e.g., Visser et al. 2004; Memmott et al. 2007; Both et al. 2009) and meta-analyses using multispecies phenological data (Parmesan and Yohe 2003 or Root et al. 2003). Currently, there are new studies addressing the changes in plant phenophases (e.g., Beard et al. 2019; Piao et al. 2019; Büntgen et al. 2022). Other papers follow “old” manuscripts (Menzel et al. 2006) about phenological events and their connection with climate change have verified the current phenological response to climate change (Menzel et al. 2020).

Most phenological studies have focused on wild plant species, which can provide excellent insight into climate change in untouched nature (Renner and Zohner 2018; Büntgen et al. 2022). Nevertheless, there are also papers evaluating the phenology of managed ecosystems, i.e., field crops (Ahas et al. 2000; Estrella et al. 2009; Xiao et al. 2013) or fruit trees (Chmielewski et al. 2004). Few papers have used phenological data from wild plants and evaluated the connection between the phenology of field crops or fruit trees and farmers’ activity (Menzel et al. 2006, 2020; Chmielewski et al. 2004). Generally, the phenological phase shifts to earlier times by several days per decade and is different for various species or crops (Renner and Zohner 2018; Büntgen et al. 2022; Menzel et al. 2020; Xiao et al. 2013).

Phenological changes still contribute to temperature and climate change; most studies rely on the fact that climate and mainly temperature conditions in the preceding months or weeks before phenophase events critically impact the timing of phenophase events (Thackeray et al. 2016; Rosenzweig et al. 2007). For example, in the UK, the first flowering dates (from 1952 to 2019) have highly negative correlations with the mean January–April maximum, mean and minimum temperatures (Büntgen et al. 2022). Although there are different site conditions for different plants (different seasons, topographies or plant traits), the impacts of climate change (and the sensitivity of plants to temperature) are still the most important aspect of phenological changes (e.g., Menzel et al. 2020).

Annually recurring events in wild plant species are called true phases because environmental factors, predominantly climate, exclusively trigger their onset. Events in agricultural species driven by farmers’ activities, such as sowing and harvesting, are known as false phases (Schnelle 1955). However, Menzel et al. (2006) stated that the phase after a false phase (e.g., emergence after sowing), although strictly defined as a true phase, is strongly related to its preceding false phase. These false phases are driven by other factors and not only by climate conditions (Menzel et al. 2020), which indicates that the true agricultural phases could be driven by other factors in addition to climate. Especially in agriculture, analyzing the evidence of change and its attribution is more complicated because farmers adapt to those climatic changes and concurrently alter their crop production (Menzel et al. 2006). Additionally, for example, the modest number of effective growing days challenges farmers’ capacity to cope with climatic constraints (Trnka et al. 2011). Nevertheless, the so-called false phases also changed the timing; the accelerated timing of sowing in spring and autumn was detected for active farmers in central Europe, Germany, Austria, and Switzerland (Menzel et al. 2020). The timing of winter wheat sowing in autumn was also evaluated in China (Xiao et al. 2013).

Since there are lots of different phenological trends, mainly within the field crop species, we decided to evaluate the differences among field crops and wild species. Within this study, we use as many phenological dates of herbs, shrubs, trees, and field crops as possible for various sites in the Czech Republic and for a long-term period (1961–2021). Our central hypothesis resulted from the assumption that the phenological trends of herbs, shrubs, trees, and field crops should differ from each other because different conditions influence them; mainly, the timing of field crops is affected not only by climate but also by the activity of farmers and their judgment when sowing starts or interferes with growth, which can influence the timing of phenophases. To verify this assumption, we focused on the following aims: (1) to calculate the trends in the phenological development of winter wheat as a representative field crop and trees, shrubs and herbs as representative wild species in the period 1961–2021 at various sites of the Czech Republic; (2) to assess which meteorological parameters influence phenological onset; and (3) to evaluate the correlations among specific species in detail to determine the differences over time.

## Materials and methods

**Fig. 1:**
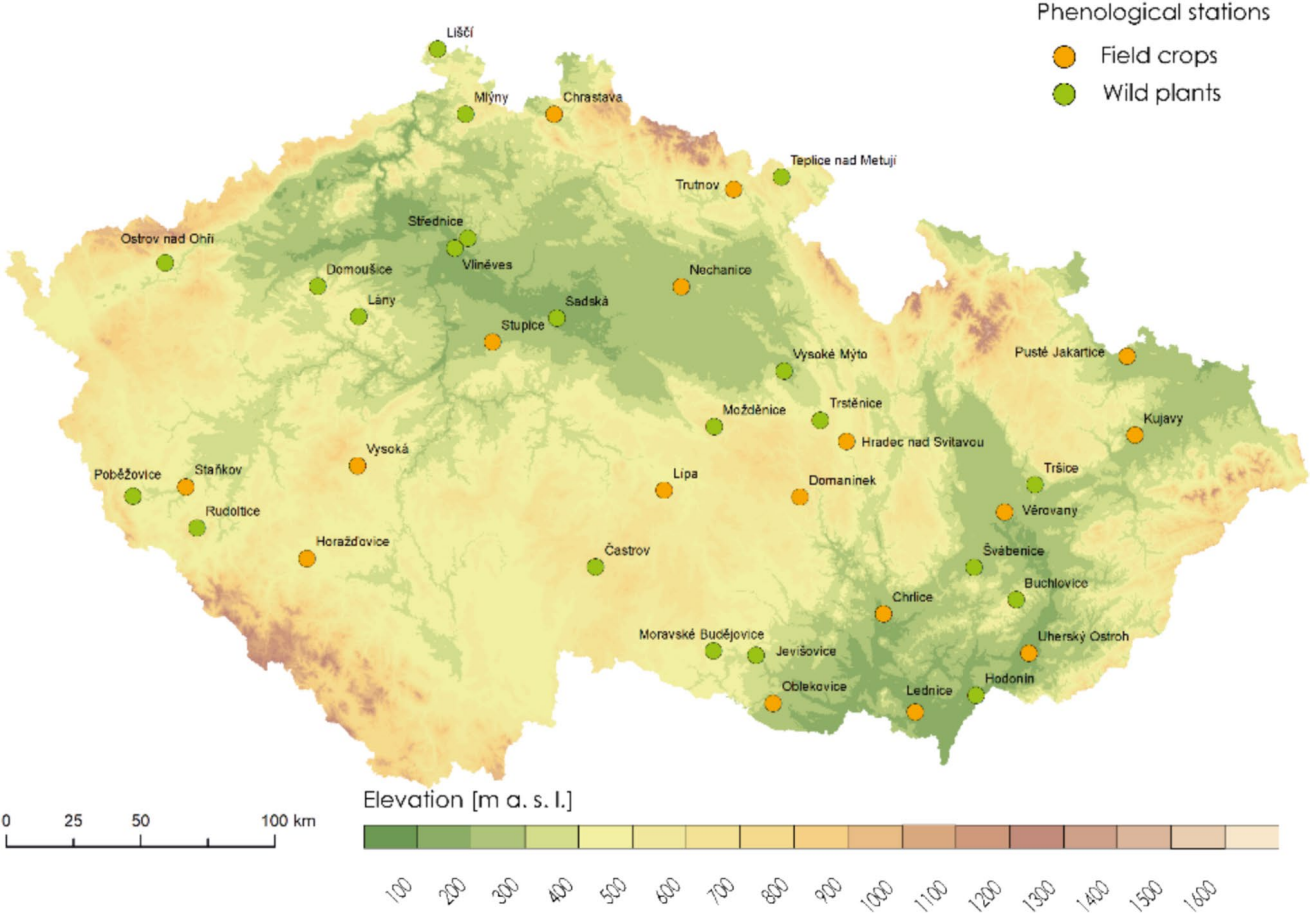
The map with phenological experimental sites – green dots indicate sites with observations of trees, shrubs and herbs; orange dots indicate sites with observations of winter wheat (*T. aestivum*) phenology

The phenological data were collected at various stations within the Czech Republic from 1961 to 2021. The observations of trees, shrubs, and herbs were performed by the Czech Hydrometeorological Institute (CHMI) and were obtained from the CHMI databases PHENODATA and CISTA. CHMI has maintained a network of phenological stations recording the phenological stages of wild plants following CHMI methodological instructions (Anonymous 2009; Coufal et al. 2004). Observations were performed continuously without any gaps for 2 trees (lime tree - *Tilia cordata*, Norway maple - *Acer platanoides*), 2 shrubs (blackthorn - *Prunus spinosa*, common hazel - *Corylus avellana*) and 1 herb (oxeye daisy - *Leucanthemum vulgare*) at 21 experimental sites throughout the Czech Republic (Table 1; Fig. 1). Two phenological phases were observed: BBCH11 (when first leaves were visible) for *A. platanoides* and *C. avellana* and BBCH61 (the start of flowering) for *T. cordata*, *P. spinosa* and *L. vulgare*. The highest number of observations occurred in areas with altitudes of 300–499 m asl, followed by lower areas (0–299 m asl), and the lowest number of observations occurred at higher altitudes (500–750 m asl).

**Table 1:** Overview of available phenological data for trees, shrubs and herbs. All observations were performed from 1961–2021

	Phenophase	Species	Localities
0–299 m asl	Start of flowers	*Tilia cordata*, *Prunus spinosa*, *Leucanthemum vulgare*	Hodonín, Vlíněves, Sadská, Buchlovice, Vysoké Mýto, Střednice, Tršice, Švábenice
	Start of leaves	*Acer platanoides*, *Corylus avellana*	Hodonín, Tršice
300–499 m asl	Start of flowers	*Tilia cordata*, *Prunus spinosa*, *Leucanthemum vulgare*	Poběžovice, Trstěnice, Morvaské Budějovice, Teplice nad Metují, Domoušice, Liščí, Jevišovice, Mlýny, Lány, Teplice nad Metují
	Start of leaves	*Acer platanoides*, *Corylus avellana*	Poběžovice, Trstěnice, Teplice nad Metují, Domoušice, Liščí, Mlýny, Jevišovice
500–750 m asl	Start of flowers	*Prunus spinosa*, *Leucanthemum vulgare*	Častrov, Možděnice
	Start of leaves	*Corylus avellana*	Častrov, Možděnice

Phenophases of field crops (winter wheat – *T. aestivum*) were monitored by the Central Institute for Supervising and Testing in Agriculture; observations were performed at small-plot experimental stations of registered winter wheat cultivars at 17 localities. The standard cultivars have been developed and changed over the entire study period, but the standard for the variety trials was always selected based on the previous standard cultivar (such that the new cultivar fits into the same 5 classes of earliness, i.e., the “medium” class). The lists of observed standard varieties with average terms of phenophases are shown within the appendices (Online resources 1, 2 and 3). The standards of wheat cultivars have never been bred for their phenological parameters in the Czech Republic, and also, on average, the differences in each year among all observed varieties are not more than 5 to 7 days. Three phenological phases were observed for *T. aestivum*: BBCH31 (jointing – first node at least 1 cm above node 1), BBCH51 (heading – beginning of heading) and BBCH89 (ripening – fully ripe hardened grains). The observations were performed in the early 1990s at most of the experimental stations; for a few stations, the observations were performed since 1961 for phenophase heading and ripening, and we also continued to elaborate on the stations where the observations started in 2000 to observe the changes in the last two decades (Table 2). The experimental sites of winter wheat and trees, shrubs and herbs are not at the same locations but span the entirety of the Czech Republic (because the observations were performed by two different institutions). Nevertheless, the sites could be categorized by altitude into three areas, as described in Tables 1 and 2, and based on these areas, the changes and trends in phenophases were evaluated.

**Table 2:** Overview of available data for winter wheat (*T. aestivum*) phenology. The year monitoring began is given for each experimental site. All observations ended in 2021

	Phenophase	Localities
0–299 m asl	Jointing	1984: Věrovany; 1992: Kujavy, Nechanice; 1993: Čáslav; 1994: Chrlice, Lednice, Oblekovice, Pusté Jakartice, Uherský Ostroh; 1995: Branišovice; 1997: Hrubčice; 2000: Žabčice, Stupice, Úhřetice
	Heading	1961: Chrlice, Oblekovice; 1963: Uherský Ostroh; 1969: Pusté Jakartice, Věrovany; 1976: Lednice; 1992–1995: Kujavy, Nechanice, Čáslav, Branišovice, Hrubčice; 2000: Žabčice, Úhřetice
	Ripening	1961: Chrlice, Oblekovice; 1963: Uherský Ostroh; 1969: Pusté Jakartice, Věrovany; 1976: Lednice; 1992–1995: Kujavy, Nechanice, Čáslav, Branišovice, Hrubčice; 2000: Žabčice, Úhřetice
300–499 m asl	Jointing	1992: Hradec nad Svitavou, Trutnov; 1993: Jaroměřice, Staňkov; 1994: Horažďovice, Chrastava
	Heading	1973: Horažďovice; 1977: Chrastava; Hradec nad Svitavou, Trutnov; 1993: Jaroměřice, Staňkov
	Ripening	1973: Horažďovice; 1977: Chrastava; Hradec nad Svitavou, Trutnov; 1993: Jaroměřice, Staňkov
500–750 m asl	Jointing	1992: Vysoká; 1994: Lípa, Domanínek
	Heading	1961: Lípa, Domanínek; 1992: Vysoká
	Ripening	1961: Lípa, Domanínek; 1992: Vysoká

Meteorological data for this study were prepared by interpolation of stations measurements of Czech Hydrometeorological Institute. The interpolation procedure employed a detailed digital terrain model in combination with meteorological data from 268 climatological stations and 787 precipitation stations in the Czech Republic (the average, maximum, minimum air temperature and precipitation were used). The daily data from each station were quality-controlled, homogenized (Štěpánek et al. 2013; Squintu et al. 2020), and subsequently interpolated by the regression kriging method using geographical coordinates, elevation, and other terrain characteristics as predictors into gridded data with a spatial resolution of 500 m The relationship between the predictors and a predictand was estimated for each meteorological variable and each day individually, and only predictors that were significant for the regression were used for the final estimates. For each of the cases, the best type of semi-variogram was assessed. The R software was used for calculations (specifically the packages rgdale and raster; Bivand et al. 2023; Hijmans 2024).

Individual climate variables were averaged for months (February to May), for pairs of months, or for longer periods. The abbreviations for each month were denoted as follows: F for February, M for March, A for April, and M for May. Then, their combinations were used for pairs and longer periods as follows: FM for February and March; MA for March and April, and AM for April and May; and for longer periods, FMA for February, March, and April; MAM for March, April, and May. The thus averaged values of the climate variables (minimum, maximum, average temperature, and precipitation) were used for further processing.

To better understand the relationships between field crops and wild species, specific phenophases and species were selected, and their correlation coefficients were calculated over 20-year periods in succession, i.e., for the periods of 1961–1980, 1962–1981, etc., until the last period of 2002–2021. For these analyses, the heading phenophase of winter wheat was chosen and compared with the start of the flower phenophase of *L. vulgare*. These phenophases and this herb species were chosen according to the availability of data; for both the heading phenophase of winter wheat and the flowering of the herb, it was possible to work with long time series from 1961 (for altitudes 500–750 m asl and 0–299 m asl) or from 1973 (for mid-altitudes 300–499 m asl). The actual dates of these phenophases are similar in time (the phenophases are, on average, 9 days apart at the lower and middle altitudes and 13 days apart at higher altitudes), which means these two species’ phenophases could reflect the same/similar climate conditions and show the specific differences between crop (winter wheat) and wild species (herb).

Correlation coefficients (r, we used the parametric Pearson coefficient) were used as the primary indicators of the strength of the relationship between the given variables. The trend is representing the slope of the linear regression between the date of the phenological date and the year. Any significance in the observed trends (for both the climatological and phenological parameters) was assessed using a t test. Additionally, we used 20-year subsamples (e.g., 1961–1980, 1962–1981, etc.) for the calculation of the running correlation to see the variability of the correlation among the phenological dates and climate parameters. All the tests were performed with the statistical/programming tool R 3.6.1. (R, http://www.climahom.eu/AnClim.html, 2024) and with AnClim software for removing potential outliers in the phenological series (Stepanek, 2008).

## Results

### Average phenological terms

The average dates of the phenological phases cover the spring period, namely, from the start of leaf development in the trees (end of March/beginning of April) to the start of the ripening of *T. aestivum* (in July). The earliest phenophases, which occur in late March/early April, are the start of leafing (*A. platanoides* and *C. avellana*), the start of flowering (*P. spinosa*) and jointing (*T. aestivum*). The start of flowering of herbs (*L. vulgare*) and heading (*T. aestivum*) also occur at similar times. Two phenophases that do not coincide with the others follow, namely, the start of flowering (*T. cordata*) and ripening (*T. aestivum*) (Table 3).

**Table 3:** Average terms of all observed phenophases for crop plants (winter wheat—*T. aestivum*) and for wild species—lime tree (*T. cordata*), Norway maple (*A. platanoides*), blackthorn (*P. spinosa*), common hazel (*C. avellana)* and oxeye daisy (*L. vulgare)*

		Triticum aestivum		Tilia cordata	Prunus spinosa	Leucanthemum vulgare	Acer platanoides	Corylus avellana
	Jointing	Heading	Ripening	Start of flowers			Start of leaves	
0–299 m asl	109	146	200	167	109	138	98	105
300–499 m asl	112	151	210	184	114	148	116	107
500–750 m asl	120	156	217	No data	120	146	No data	116

### Phenological trends

The dates of spring phenophases are shifted to earlier times for both wild plants and field crops. For wild plants, most of the trends are statistically significant, with only a few species from a small number of sites exhibiting nonsignificant changes (of the 36 study areas, 30 exhibited a significant shift and 6 showed nonsignificant trends, but even those with a nonsignificant trend exhibited a shift to an earlier time). Winter wheat, on the other hand, showed both significant and nonsignificant shifts; of the 66 study areas, 36 always showed a significant trend to an earlier time, and the remaining 30 did not show changes, with a change in phenophase date to an earlier time and, in two cases, to a later time.

The statistically significant phenological trends of winter wheat differ at different altitudes. The trends of the phenophase of jointing showed the greatest variability with altitude (in the lowlands, there was a shift of 4.5 days per decade, while in the middle and higher altitudes, there was 6.9 and 5.7 days per decade, respectively). On the other hand, the heading phenophase indicates stable changes in the shifting trend regardless of altitude (3.3–4.0 days per decade), and the ripening phenophase again shows slightly more variability (3.3–4.8 days per decade; Table 4). In the case of wild plants, different trends were evident at lower, middle, and higher altitudes. The phenophases of the beginning of flowering and the beginning of leafing (trees, shrubs, and herbs) were significant at lower and middle altitudes (a shift of 2.7 and 2.9 days per decade to earlier times, respectively). Conversely, at higher elevations, the phenophase dates are shifted by 4.1 days per decade.

**Table 4:** Statistically significant trends (average trends for all stations, minimal and maximal trends at given stations) for each phenophase at various altitudes for 3 phenophases of winter wheat (*T. aestivum*) and 2 phenophases of trees, shrubs, and herbs

	Winter wheat	AVERAGE trend/decade/ days	MIN trend/ decade/days	MAX trend/ decade/days
0–299 m asl	Jointing	−4.5**	−2.8**	−6.0**
	Heading	−3.3***	−2.6***	−4.1***
	Ripening	−4.4***	−3.1***	−5.9***
300–499 m asl	Jointing	−6.9***	−6.9***	−6.9***
	Heading	−3.6***	−3.0***	−4.1***
	Ripening	−4.8**	−3.2*	−5.7**
500–750 m asl	Jointing	−5.7**	−5.3**	−5.9***
	Heading	−4.0***	−3.7***	−4.2***
	Ripening	−3.3**	−3.1*	−3.4***
	Trees, shrubs, herbs	Trend/decade/ days	MIN. trend/ decade/days	MAX. trend/‘ decade/MAX
0–299 m asl	Start of flowering (tree, shrub, herb)	−3.0***	−1.8***	−4.2***
	Start of leaves (shrub)	−2.5***	−2.5***	−2.5***
300–499 m asl	Start of flowering (tree, shrub, herb)	−3.1***	−2.2***	−4.8***
	Start of leaves (tree, shrub)	−2.7***	−2.0**	−4.5***
500–750 m asl	Start of flowering (shrub, herb)	−3.6***	−3.5***	−3.6***
	Start of leaves (shrub)	−4.7***	−4.6***	−4.8***

* Significant at α = 0.05; ** significant at α = 0.01; *** significant at α = 0.001%

**Fig. 2:**
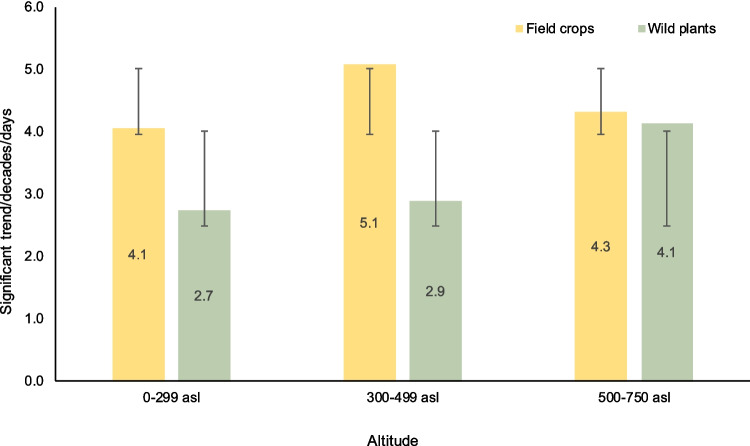
Average statistically significant phenological trends with standard deviations of winter wheat (*T. aestivum*) and wild plants (average values for trees, shrubs and herbs) at various altitudes and during the period of 1961–2021

**Fig. 3:**
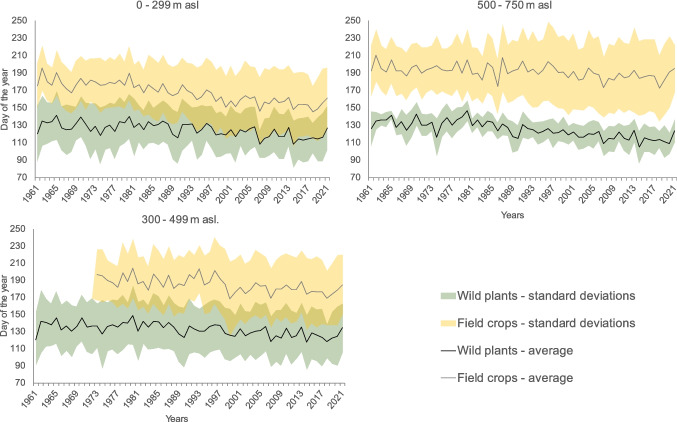
Average phenological terms for all observed phenological phases of field crops and wild-growing species between 1961 and 2021 with standard deviations

Thus, we generally observed different trends between field crops and wild species at lower and middle elevations (Fig. 2). Compared with those of the wild species, the spring phenophase of winter wheat at lower elevations shifted by 4.1 (which means faster shift by 1.4 days according to wild species) and at middle elevations the shift was by 5.1 days (which is faster shift by 2.2 days according to the wild species). At the same time, a similar shift to earlier times for both (field crops and wild species) prevails at higher elevations. The spring phenophases of winter wheat and wild species (herbs, shrubs) advanced by 4.3 and 4.1 days per decade, respectively (Table 4). The changing phenological manifestations of field crops, specifically since the late 1980s and the start of the 1990s, are visible at low and middle elevations. The average phenological terms for both field crops and wild species showed advancing trends to earlier times. However, for field crops, specific phenological accelerations are not visible at higher elevations (Fig. 3).

### Phenological correlations

The correlations between the spring phenophases of winter wheat and wild species and climate parameters also differed. First, the individual phenophases of winter wheat correlate differently with climatic parameters. The strongest link with climatic variables is evident for the heading phenophase. The correlations with the mean and maximum temperatures (in the months preceding the phenophase) were the strongest and most statistically significant (mean value *r* = 0.8). A statistically significant correlation was also determined for the minimum temperature (*r* = 0.7) and for precipitation at most sites (no correlation was found at 3 stations, and the remaining 18 sites had an average significant *r* = 0.5). The next two phenophases (jointing and ripening) were less strongly correlated with precipitation. In the case of jointing, a statistically significant correlation was shown at only 3 stations (of 21 total), and for ripening, significant correlation was observed at 7 stations. However, for these two phenophases, a significant association was shown with the mean and maximum temperatures (*r* = 0.7 for jointing and *r* = 0.6 for ripening) (Table 5). Correlations with temperature were confirmed for all three phenophases for temperature (or precipitation) in the months preceding the phenophase—February, March and April for jointing and March, April and May for heading and ripening.

**Table 5:** Correlation coefficients for terms of phenophases and climate parameters (T AVG, T MAX, T MIN – average, maximal and minimal temperature and precipitation indicated as PREC)

		T AVG	T MAX	T MIN	PREC
Altitude	Phenophases/ species	Correlation coefficients			
0–299 m asl	Jointing (winter wheat)	0.7***/FMA	0.7***/FMA	0.7***/FMA	0.4*/AM
	Heading (winter wheat)	0.8***/MAM	0.8***/MAM	0.7***/MAM	0.5*/AM
	Ripening (winter wheat)	0.6***/MAM	0.6**/MAM	0.6**/MAM	0.5*/MAM
	Start of flowering (tree, shrub, herb)	0.7**/FMA	0.8***/MA	0.6**MA	NA
	Start of leafing (tree, shrub)	0.7***/FMA	0.6**/FMA	0.7***/FMA	NA
300–499 m asl	Jointing (winter wheat)	0.7**/FMA	0.7**/FMA	0.7***/FMA	0.4*/AM
	Heading (winter wheat)	0.9***/MAM	0.9***/MAM	0.7***/MAM	0.5*/AM
	Ripening (winter wheat)	0.6**/MAM	0.6***/MAM	0.6**/MAM	NA
	Start of flowering (tree, shrub, herb)	0.7***/MAM	0.8***/MAM	0.6**/MAM	NA
	Start of leaves (tree)	0.7***/MA	0.8***/MA	0.6**/MA	0.3*/MA
500–750 m asl	Jointing (winter wheat)	0.8***/FMA	0.7***/FMA	0.7***/FMA	0.4*/AM
	Heading (winter wheat)	0.9***/MAM	0.9***/MAM	0.8***/MAM	0.5*/AM
	Ripening (winter wheat)	0.6**/MAM	0.6**/MAM	0.6**/MAM	NA
	Start of flowering (shrub, herb)	0.7***/MAM	0.8***/MAM	0.6**/MAM	NA
	Start of leaves (shrub)	0.7***/MAM	0.7***/MAM	0.6**/MAM	NA

* Significant at α = 0.05; ** significant at α = 0.01; *** significant at α = 0.001%

For wild species, the correlation with maximum temperature in the months preceding the phenophase was notable (*r* = 0.8 for the start of flowers and *r* = 0.7 for the start of leaves). There was also a clear correlation with the mean and minimum temperatures. On the other hand, there was almost no correlation with precipitation; only at the start of leafing phenophase at middle altitudes (300–499 m asl) was there a statistically significant correlation, but the correlation coefficient was low (*r* = 0.3), and the correlation coefficient was low at only three locations.

**Fig. 4:**
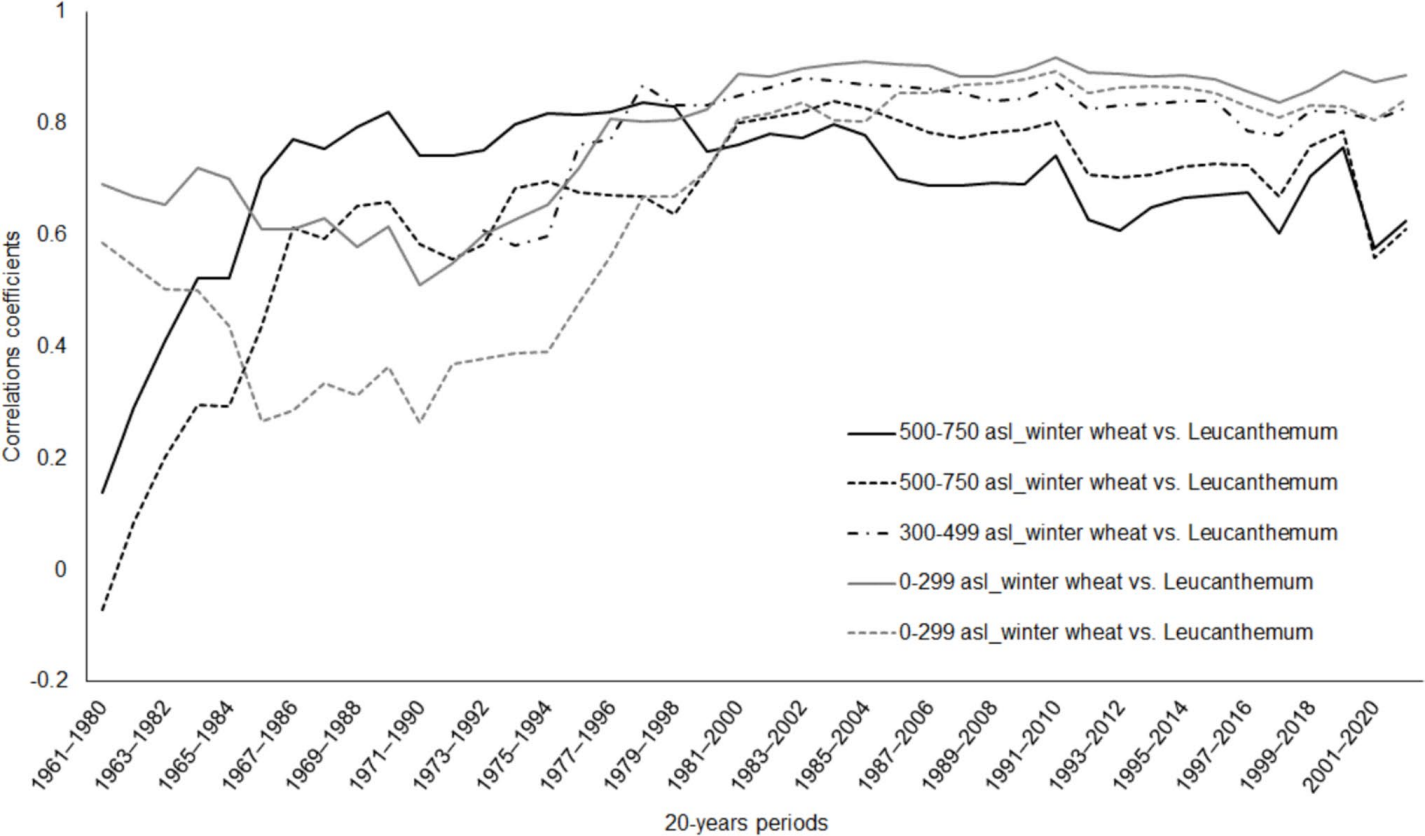
Correlation coefficients at 20-year periods (1961–1980, 1962–1981, etc.) for winter wheat (*Triticum aestivum*) and ox-eye daisy (*Leucanthemum vulgare)* at two different stations within low altitudes (0–299 m asl), at one station within middle altitudes (300–499 m asl) and at two different stations at high altitudes (500–750 m asl)

The phenological phase of *T. aestivum* heading and the onset of *L. vulgare* flowering exhibit a phenological relationship over a 20-year period. At higher elevations (500–750 m asl), a significant difference in the onset of phenophases is evident in the 1960s and 1970s, when the values of the correlation coefficients are approximately zero and gradually increase. In the mid-elevations (300–499 m asl), monitoring started in 1973, and there was also a weakly noticeable decrease in the correlation only in the 1970s. A similar trend was also evident at low elevations (0–299 m asl), where a decrease in correlation was evident in the second half of the 1960s and into the 1970s (Fig. 4). Two analyses were always performed at low and high altitudes since only the heading phenophase of winter wheat was observed at two different locations.

## Discussion

The principal finding of this paper is that within the region of the Czech Republic, field crops (specifically winter wheat) are shifting to the start of the year with higher intensity than wild-growing plants (herbs, shrubs, and trees) based on observations from 1961 to 2021 at 38 locations. The difference in phenological trends was defined specifically at low and middle altitudes (0–299 m asl and 300–499 m asl), whereas at higher altitudes (500–750 m asl), the trends were similar for crops and wild plants.

Generally, the rate of phenological phases shifts to earlier times by 1–4 days per decade for the flowering of herbs and shrubs and by 2–3 days for the leaf unfolding of trees in Europe (Renner and Zohner 2018); for example, in the UK, the average phenological advancement is 5.4 days per decade (Büntgen et al. 2022) for herbs, shrubs, and trees. This observation is consistent with our results (3–4 days per decade for herbs, shrubs, and trees and two phenophases at the start of leaves and the start of flowers), which were also confirmed by a detailed phenological study from one experimental site within the Czech Republic (the average significant shift moved from 1.4 days to 2.3 days for herbs, shrubs, trees and bird populations) (Bartosova et al. 2022). An increase of 0.307 days/year (i.e., 3.1 days per decade) for vegetative phenophases or 0.223 days/year (i.e., 2.2 days per decade) for generative phenophases in Europe was also detected for spring phenophases of field crops (Menzel et al. 2020). The phenological phases of winter wheat in China accelerated by 1.1–2.7 days per decade (Xiao et al. 2013). The heading of winter wheat in US showed negative trends by 0.3 to 1.9 days per decade (from 1948 to 2004) (Hu et al. 2005). Our results showed a greater shift to earlier days; for example, phenophase jointing showed an advancing trend of up to 6–7 days in one decade at all observed altitudes (0–299, 300–499 and 500–750 m asl), and the phenological change was on average 4–5 days for all winter wheat phenophases and still greater according to other papers. On the other hand, there are also records showing the reverse trend—e.g., winter wheat showed a delay in the timing of phenological phases (by 1.5–1.7 days per decade) in China (Xiao et al. 2013). Our data also showed the opposite trend – only two experimental sites showed a delay in the timing of phenophases, but the difference was nonsignificant. We need to stress that nonsignificant phenological trends were also detected for negative trends (which means shifts to earlier times) within our data. This means there are lots of different phenological trends within the field crops. For example, for another agricultural crop, the trends also showed negative trends: in Germany, the oat phenophases advanced the onset of phenophases by 0.2 and 0.4 days per decade (for sowing and emergence) and by 1.9, 3.3 and 2.1 days per decade (for heading, yellow ripeness and harvest respectively) during the 1959 and 2009 (Siebert and Ewert 2012). In western Germany, the winter rye and winter rapeseed were also elaborated, and phenological trends showed shifting by 4 to 20 days to the earlier dates in the period 1960–2013 for true and false phenophases; nevertheless, some phenophases (specifically emergence) showed no trends and some positive trends (specifically harvest) (Rezaei et al. 2017). Within the Czech Republic the winter oilseed rape phenological trends were also studied and showed negative trends by 10.7–15.6 days (during 1991–2012) (Hájková et al. 2021). Quite noticeable negative trends were detected over the Iberian Peninsula where specifically two phenophases (flag leaf sheath swollen and flowering date) of wheat and oat showed shift by 1 to 3 days per year (i.e. 10–30 days per decade) (Oteros et al. 2015). So-called false phases also exhibited changes in timing; the accelerated timing of sowing in spring and autumn was determined for active farmers in central Europe (Germany, Austria and Switzerland) to be 0.116 days/year (i.e., 1.2 days per decade) and 0.109 days/year (i.e., 1.1 days per decade), respectively. Greater shifts to earlier times were detected for summer harvests, namely, 0.246 days/year (i.e., 2.5 days per decade) (Menzel et al. 2020). Furthermore, opposite trends show delays in the sowing of winter wheat during autumn in China (by 1.5 days per decade) (Xiao et al. 2013). As already mentioned, the phenological trends of already studied crop fields and various phenophases showed quite big differences. Each study connected the phenological changes with temperature trends (also our phenological trends were significantly correlated with temperature). Nevertheless, the photoperiod and vernalization is also considered to be driving factor for timing phenophases of winter wheat (Rezaei et al. 2017). But there are studies showing the differences between old cultivars with high sensitivity and new cultivars with lower sensitivity of winter wheat to the photoperiod (Motzo and Gunta 2007; Grogan et al. 2016); and is also known the winter wheat varieties vary in requirement for vernalisation (McMaster et al. 2008). Still the combination of vernalization requirements, photoperiod sensitivity and temperature requirements are considered to be driving factors for the growth of winter wheat (Rezaei et al. 2017). The water availability is also detected to be the key factor for affecting the phenology (Schwartz 2003) and also the CO_2_ concentrations were considered to slightly influence (speed up) the phenology (Kimball et al. 2002). And last but not least the farmer´s decisions play important role as was already shown in previous studies mainly for so called false-phases (sowing and harvesting) (e.g. Estrella et al. 2007). It means not only the phenological trends showed various outputs from different locations and or for different field crop. But also the different influence on phenological timing have to be considered. It was not the aim of this paper to evaluate which parameter (temperature, photoperiod, vernalization, farmer activities, etc.) play the crucial role but need to be mentioned here to show there are lots of impacts influencing the phenology of field crops resulting in different phenological trends of field crops.

Within our results, we observed a difference specifically between crops and wild species at lower and middle altitudes (where cropping plants showed a stronger phenological shift). At higher altitudes (500–750 m asl), the phenological trends were almost the same for winter wheat and shrubs and herbs, so the wild species also showed the same phenological speed as the crop plants. Very similar outputs were also recorded for forest trees and fruit trees in Switzerland, where a stronger phenological shift occurred at higher elevations (predicted mean across species and all 50 stations: −3.9 days per decade) than at lower elevations (− 2.2 days per decade) (Vitasse et al. 2018). The difference between lower and higher elevations could be explained by the different temperature trends—stronger warming during late rather than early spring (Vitasse et al. 2018)—which was not proven according to our data (the temperature trends were not different at lower, middle or higher elevations). The reason for the smaller phenological trends (in wild plants) at lower and middle elevations could be that phenophases occur early in the spring, which indicates that they could be limited by late frosts or photoperiods that decelerate the onset of phenophases. The mean number of frost risk days after the false spring onset calculated for the territory of the Czech Republic is moving in 0–10 days within the area of experimental sites with higher elevations with wild species observations. Which is less according to lower areas with 10–20 days with possible frost risk (Zahradníček et al. 2024). Nevertheless, other studies have reported stronger phenological trends at higher elevations, e.g., for trees in Poland (Chmura and Rozkowski 2002), or using phenological process-based models, the predicted bud bursting is accelerated at higher elevations (Gauzere et al. 2017). Also the winter wheat phenological trends show faster response in higher elevations (by 1.6 to 1.9 days per decade above the 500 m asl) compared to low elevations (by 0.3 to 1.6 days per decade in 273 to 357 m asl) (Hu et al. 2005).

However, the main outcome of our paper is that field crops (specifically winter wheat) show greater phenological trends than wild species. This disharmony in phenological response can be mainly explained by the influence of human farmers, who can significantly interfere with the growth of field crops. Although in this study, we evaluate phenological phases that cannot be so clearly influenced by the farmer, such as the so-called false phase (at the sowing date and harvest date), we still perceive a significant contribution of the farmer to the timing of the phenophases. However, previous studies have shown that the so-called false phases show fewer phenological trends. For example, Germany’s first hay cut showed a decreasing trend relative to that of wild-grown grass (Bock et al. 2013). Additionally, farmers’ activities (false phases such as drilling, tilling, and harvesting) showed relatively small impacts on phenophases according to the leaf unfolding and flowering data across Europe (Menzel et al. 2006). Therefore, the phenological signal in agriculture was weaker than the signal for wild-growing plant species, and the connection between agricultural phenophases and climate change was weak. There are also assumptions that eventually, agricultural management and/or cultivar choice will also be adapted to the new potential growing seasons (Menzel et al. 2020). Our findings show that agriculture’s true phenophases not only correlate with spring temperatures, as do those of wild-growing species, but also show more progressive phenological trends according to those of wild-grown species. This suggests that farmers are adapting to changing climate conditions and adapting to the timing of false phases (particularly sowing dates) and then accelerating upcoming phenological phases, not only with earlier sowing dates but also with possible earlier application of fertilizers, which can speed up growth. This is also supported by the projections that the early-spring sowing window should become longer (on average) and more stable in the continental zone (according to the Metzger zones; Trnka et al. 2011). These changes agree well with the shorter duration of snow cover and increasing spring temperatures (Brazdil et al. 2009). The same study also showed that effective global radiation and effective growing days should be lower in middle and southern Europe (especially due to drought stress). This indicates that farmers are facing new climate conditions and are trying to adapt their management and timing of false phases. Similar outcomes were also described when using the vegetation indices to define the start of the season—the coverage of arable land showed the most progressive shift to earlier dates compared to other land –coverage, such as that of grasslands, broad-leaved forests, and coniferous forests (Dížková et al. 2024, under revisions). Thus, specific phenological changes in field crops can be observed locally and over a wide area, suggesting that not only changing climate but also crop characteristics themselves, influenced by variety or breeding, may be involved in changes in the timing of phenophases.

## Conclusion

It is well known that phenological phases during the spring exhibit advancing trends, causing the earlier start of the vegetation period. Nevertheless, there are also signals that different species exhibit different phenological reactions. Our study showed clear phenological differences between field crops (represented by winter wheat and its spring phenophases) and wild plants (represented by spring phenophases of herbs, shrubs, and trees), especially at low and middle altitudes, where there were more expressive shifts in crop plants. We believe these phenological changes in crop plants are correlated with rising temperatures and influenced by farmers’ activities and their changing time management during the springtime. Our main recommendations for current and also future studies of climate change and climate change impacts are two: first, based on the results of this study, we suggest using the phenological trends of mainly wild species to clearly see the natural response of species, ecosystem, or landscape to rising temperatures (i.e. climate change); and second under current climate conditions with rising temperature during the springtime be aware of the risk of spring frosts mainly for field crops which showing fast phenological shift.

## Supplementary information

Below is the link to the electronic supplementary material.

ESM 1 (DOCX 16.5 KB)
