# Supplementary material for: Differences in phenological term changes in field crops and wild plants – do they have the same response to climate change in Central Europe?
Source: Int J Biometeorol. 2025 Jan 7;69(3):659–70. doi: 10.1007/s00484-024-02846-8 (PMC11860992; doi:10.1007/s00484-024-02846-8)
Supplement: Supplementary file 1 — (DOCX 16.5 KB) [file 484_2024_2846_MOESM1_ESM.docx]

SUPPLEMENTARY DATA

Online Resource 1: The list of observed standards varieties within the study with average terms of phenophases and standard deviations in the low elevations (0-299 m asl). DOY – day of the year. SD – standard deviation.

| Varietes | Jointing Average term/DOY | Heading Average term/DOY | Ripening Average term/DOY | Jointing SD/days | Heading SD/days | Ripening SD/days |
| --- | --- | --- | --- | --- | --- | --- |
| Diana | 113 | 155 | 204 | 7.3 | 7.3 | 9.4 |
| Pavlovická | 113 | 155 | 205 | 7.9 | 8.6 | 10.7 |
| Kaštická osinatka | 112 | 155 | 205 | 6.5 | 6.2 | 8.3 |
| Mironovská | 108 | 149 | 200 | 5.3 | 5.9 | 8.9 |
| Jubilejná | 109 | 147 | 197 | 7.1 | 5 | 8.1 |
| Iljičovka | 109 | 148 | 199 | 5.7 | 5.9 | 9.9 |
| Vala | 107 | 146 | 203 | 6.5 | 6.3 | 9.7 |
| Odra | 107 | 146 | 202 | 7.1 | 4.9 | 8.2 |
| Hana | 106 | 146 | 200 | 6.6 | 4.4 | 6.6 |
| Samanta | 107 | 140 | 197 | 7.5 | 5.1 | 5.3 |
| Bohemia | 106 | 140 | 197 | 8.9 | 6.2 | 6.7 |

Online Resource 2: The list of observed standards varieties within the study with average terms of phenophases and standard deviations in the middle elevations (300-499 m asl). DOY – day of the year. SD – standard deviation.

| Varietes | Jointing Average term | Heading Average term | Ripening Average term | Jointing SD/days | Heading SD/days | Ripening SD/days |
| --- | --- | --- | --- | --- | --- | --- |
| Diana | 115 | 162 | 217 | 5.5 | 5.2 | 10.9 |
| Pavlovická | 115 | 162 | 216 | 7.2 | 4.3 | 11.2 |
| Kaštická osinatka | 115 | 161 | 217 | 4.3 | 5.3 | 13.8 |
| Mironovská | 113 | 160 | 217 | 3.2 | 4.5 | 12.4 |
| Jubilejná | 114 | 160 | 215 | 6.6 | 3.3 | 13.9 |
| Iljičovka | 113 | 160 | 217 | 5.9 | 5.1 | 14.2 |
| Vala | 112 | 156 | 217 | 4.8 | 6.4 | 9.6 |
| Odra | 112 | 155 | 217 | 7.8 | 5.2 | 8.8 |
| Hana | 112 | 154 | 220 | 5.8 | 4.5 | 3.3 |
| Samanta | 111 | 150 | 209 | 7.5 | 5.2 | 4.7 |
| Bohemia | 111 | 150 | 210 | 4.6 | 5.7 | 5.6 |

Online Resource 3: The list of observed standards varieties within the study with average terms of phenophases and standard deviations in the high elevations (500-750 m asl). DOY – day of the year. SD – standard deviation.

| Varietes | Jointing Average term | Heading Average term | Ripening Average term | Jointing SD/days | Heading SD/days | Ripening SD/days |
| --- | --- | --- | --- | --- | --- | --- |
| Diana | 122 | 165 | 222 | 5.5 | 7.3 | 8.7 |
| Pavlovická | 122 | 166 | 223 | 4.9 | 7.1 | 9 |
| Kaštická osinatka | 122 | 166 | 224 | 6.3 | 7.6 | 7.6 |
| Mironovská | 121 | 162 | 223 | 8.2 | 6.1 | 10.8 |
| Jubilejná | 120 | 162 | 222 | 7.3 | 5.7 | 6.7 |
| Iljičovka | 121 | 161 | 223 | 6.1 | 5.5 | 8.7 |
| Vala | 119 | 156 | 225 | 5.9 | 6.1 | 13.3 |
| Odra | 119 | 158 | 225 | 5.3 | 6.5 | 13.6 |
| Hana | 119 | 156 | 226 | 4.9 | 6.5 | 12.1 |
| Samanta | 118 | 151 | 217 | 6.7 | 5.8 | 11.7 |
| Bohemia | 118 | 151 | 217 | 6.3 | 5.9 | 8.5 |
